# Air enema reduction versus hydrostatic enema reduction for intussusceptions in children: A systematic review and meta-analysis

**DOI:** 10.1371/journal.pone.0297985

**Published:** 2024-03-18

**Authors:** Lan Liu, Ling Zhang, Yifan Fang, Yingying Yang, Wen You, Jianxi Bai, Bing Zhang, Siqi Xie, Yuanyuan Fang

**Affiliations:** Department of Pediatric Surgery, Fujian Children’s Hospital (Fujian Branch of Shanghai Children’s Medical Center), College of Clinical Medicine for Obstetrics & Gynecology and Pediatrics, Fujian Medical University, Fuzhou, China; Hacettepe University: Hacettepe Universitesi, TURKEY

## Abstract

**Objectives:**

We conducted a comprehensive meta-analysis to compare the effectiveness and safety of fluoroscopy-guided air enema reduction (FGAR) and ultrasound-guided hydrostatic enema reduction (UGHR) for the treatment of intussusception in pediatric patients.

**Methods:**

A systematic review and meta-analysis were conducted on retrospective studies obtained from various databases, including PUBMED, MEDLINE, Cochrane, Google Scholar, China National Knowledge Infrastructure (CNKI), WanFang, and VIP Database. The search included publications from January 1, 2003, to March 31, 2023, with the last search done on Jan 15, 2023.

**Results:**

We included 49 randomized controlled studies and retrospective cohort studies involving a total of 9,391 patients, with 4,841 in the UGHR and 4,550 in the FGAR. Specifically, UGHR exhibited a significantly shorter time to reduction (WMD = -4.183, 95% CI = (-5.402, -2.964), P < 0.001), a higher rate of successful reduction (RR = 1.128, 95% CI = (1.099, 1.157), P < 0.001), and a reduced length of hospital stay (WMD = -1.215, 95% CI = (-1.58, -0.85), P < 0.001). Furthermore, UGHR repositioning was associated with a diminished overall complication rate (RR = 0.296, 95% CI = (0.225, 0.389), P < 0.001) and a lowered incidence of perforation (RR = 0.405, 95% CI = (0.244, 0.670), P < 0.001).

**Conclusion:**

UGHR offers the benefits of being non-radioactive, achieving a shorter reduction time, demonstrating a higher success rate in repositioning in particular, resulting in a reduced length of postoperative hospital stay, and yielding a lower overall incidence of postoperative complications, including a reduced risk of associated perforations.

## Introductions

Intussusception stands as the most prevalent etiology of intestinal obstruction in pediatric patients. A substantial majority, approximately 75–90%, exhibit no identifiable cause and are classified as idiopathic intussusception [1–4]. This condition primarily affects the small intestine, with infrequent occurrences in the large intestine [5]. Clinical presentation typically encompasses symptoms such as abdominal pain, vomiting, and hematochezia, although the classic triad of symptoms is encountered in less than 25% of cases [6,7]. Historically, fluoroscopy-guided air enema reduction (FGAR) has served as the primary therapeutic modality for intussusception. Its prominence stems from the demonstrated efficacy and safety of enema decompression established during the 1940s and 1950s. In recent years, ultrasound-guided hydrostatic enema reduction (UGHR) has gained traction as a non-invasive, radiation-free imaging technique [8–10]. The advent of UGHR in clinical practice traces its origins back to 1982 when Kim et al. [11] first reported successful reduction of ileocolonic intussusception using warm saline enema under real-time ultrasound guidance. This approach has progressively gained popularity and involves ultrasound confirmation of the intussusception’s location. A predetermined initial pressure is established, followed by ultrasound-guided injection of warm saline into the intestinal tract. Successful reduction is verified when saline flows into the intestinal tract from the ileocecal region, resulting in the manifestation of characteristic signs such as the “crab claw sign” and “honeycomb sign” [12]. Although numerous studies have indicated that UGHR has advantages such as a higher success rate of resetting, greater safety, and radiation-free procedures, these merits are considered worthy of implementation in clinical practice. However, some studies also suggest that FGAR, as a traditional treatment method, remains practical in clinical settings due to its simplicity, ease of execution, and shorter learning curve. Besides, despite the burgeoning utilization of UGHR, a notable gap persists in terms of comprehensive, large-scale systematic comparisons and analyses assessing the efficacy, safety, and long-term prognostic implications of FGAR versus UGHR. We conducted a comprehensive meta-analysis comparing the efficacy and safety of air enema reduction and hydrostatic enema reduction for the treatment of childhood intussusception. Through an extensive literature search and rigorous clinical data analysis, our study aims to present a more secure and dependable therapeutic alternative for children with intussusception, thereby furnishing clinicians with compelling diagnostic and treatment evidence.

## Methods

Reporting followed the Preferred Reporting Items for Systematic Reviews and Meta-Analysis (PRISMA) guidelines [13] (S1 Checklist). We registered the study on PROSPERO, of which the registration number was CRD42023414518.

We conducted a systematic review of studies published in PUBMED, Google Scholar, MEDLINE, Cochrane, China National Knowledge Infrastructure (CNKI), Wanfang Database, VIP Database. The search has a limit on date from Jan 1, 2003 to Mar 31, 2023, with the last search done on Jan 15, 2023. No publication restrictions or study design filters were applied. We formulated the search strategy, inclusion criteria and exclusion criteria according to the PICOS principles: (1) Type of study: randomized or non-randomized controlled trial, with the language limited to Chinese and English; (2) Participants of the study: pediatric patients (aged <18 years) who underwent enemas due to intussusception; (3) Interventions adopted: fluoroscopic air enema or ultrasound-guided saline enemas were used; (4) The main outcome indicators: time to reset, success rate of reset, recurrence rate, and occurrence of postoperative complications; (5)The search strategy for those databases was as follows: ((enema [Title/Abstract]) AND (intussusceptions [Title/Abstract])), hydrostatic enema for intussusceptions, ((enema [Title/Abstract]) AND (intussusceptions [Title/Abstract])) AND (ultrasound [Title/Abstract]), Reference lists from related articles were also scanned to broaden the search. A hand search was performed in all six databases.

Inclusion criteria were applied as follows: (1) confirmation of intussusception diagnosis; (2) subjects aged below 18 years; (3) availability of relevant outcome measures, such as patient numbers, study design, clinical symptomatology, reset success rates, complications, and recurrence; (4) provision of suitable statistical estimates or counts; and (5) comparative investigations involving both fluoroscopy-guided air enema reduction and ultrasound-guided hydrostatic enema reduction.

Exclusion criteria were applied as follows: (1) case reports involving fewer than five cases; (2) subjects exceeding 18 years of age; (3) articles categorized as reviews or meta-analyses; (4) conference abstracts; (5) articles with insufficient data; (6) cases included that did not pertain to acute intussusception or were combined with secondary intussusception; and (7) studies lacking a direct comparison between fluoroscopy-guided air enema reduction and ultrasound-guided hydrostatic enema reduction.

The following data were extracted: the first author’s name, year of publication, study type, mean age, gender distribution, patient count, primary clinical symptoms, time required for reduction, reset pressure applied, reset success rate, duration of occult blood in stool, time until recovery of bowel function, length of hospital stay, recurrence rate, and complications.

The quality assessment of randomized controlled studies (RCTs) was conducted using the Cochrane Collaboration’s Risk of Bias tool [14]. Only studies with low or unclear risk of overall bias were included in the meta-analysis. Non-randomized studies underwent assessment with the Newcastle-Ottawa Scale (NOS) [15]. The NOS score, ranging from 0 to 9 stars, evaluates studies across three categories: selection, comparability, and outcome/exposure. Studies with a NOS score of ≥6 stars were deemed high quality and incorporated into our analysis. The literature retrieval and data collection were to be carried out by at least two researchers. They independently read the titles and abstracts of the literature, excluding those that were not relevant to the content of this study. Subsequently, they will carefully read the full texts according to inclusion and exclusion criteria, extracting relevant information. In case of disagreements, resolution will be sought through negotiation, or a third researcher may be consulted for assistance in making a judgment.

Statistical analysis was conducted by STATA version 16.0 and RevMan version 5.2. Relative risk (RR) was applied for dichotomous variables, and weighted mean difference (WMD) was applied for continuous variables. Some study outcomes were reported as medians with ranges or mid-quartiles with ranges. According to the methods introduced by Luo et al. [16] and Wan et al. [17], those data were converted to means with deviations, thus the results for each group are presented as the mean ± standard deviation (x± s). The I^2^ statistic was used to test the degrees of heterogeneity, the P-value of I^2^ < 0.05 was used to indicate high heterogeneity and vice versa. The random-effects model was applied to pool the high heterogeneity results and the fixed-effects model was used for low heterogeneity (P-value of I^2^ > 0.05; Table 2A and 2B). Begg’s Test and Egger’s Test were performed to assess the risk of bias (Table 3), while Begg’s funnel plots were applied. P < 0.05 was considered to be statistically significant in the text.

## Results

**Fig 1:**
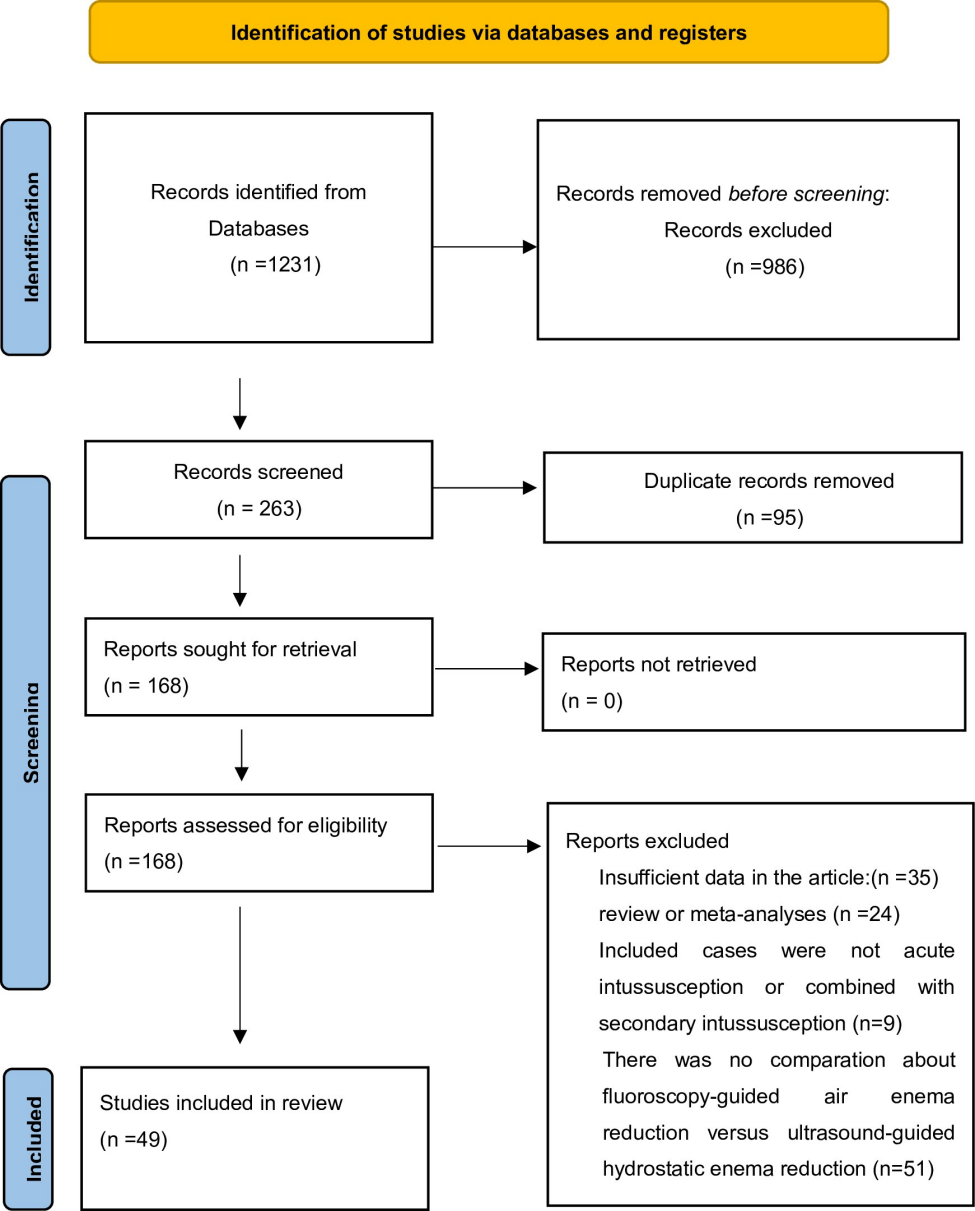
Flow diagram representing the selection of study.

We initially identified 1231 articles through our comprehensive literature search. Prior to screening, 986 records were expunged from consideration. Subsequently, after the removal of duplicate entries, an additional 119 records were excluded following a meticulous full-text review, as they failed to satisfy our predefined inclusion criteria (Fig 1). Ultimately, our analysis encompassed a total of 49 studies mostly from the Asia and Europe, involving 9391 patients, with 4841 in the ultrasound-guided hydrostatic enema reduction group (UGHR) and 4550 in the fluoroscopy-guided air enema reduction group (FGAR).

### Characteristics and risk of bias of included studies

The baseline characteristics of the 49 records, including first author, publication year, study type, number of patients, male/female sex ratio, and age of operation, are presented in Table 1.The NOS scores ranged from 6 to 8 stars, reflecting the quality of the non-randomized controlled studies (case-control and cohort studies) (S1 Table), and S1A and S1B Fig presents the Cochrane Collaboration’s Risk of Bias Tool for the randomized controlled studies (RCTs) that were judged to have a low risk of bias. Table 2(A) and 2(B) show the overall analyses for dichotomous and continuous variables, respectively.

**Table 1:** Baseline characteristics of 49 records with 9391 patients enrolled in the meta-analysis.

Name	Year	Study type	Number of patients		Gender(male/female)		Age(m)	
			U	F	U	F	U	F
Wang et al [18]	2013	RCT	46	46	38/8	40/6	15±5.04	14.16±10.2
Zhang et al [19]	2014	RCT	64	64	42/22	40/24	5.89±1.12	6.03±1.34
Guo et al [20]	2014	R	352	230	198/154	152/78	3–132	3–60
Yi et al [21]	2015	RCT	39	39	25/14	26/13	24.24±8.16	23.76±10.68
Zhong et al [22]	2015	RCT	44	40	‘32/12	27/13	14.4±1.32	10.8±1.44
Wu et al [23]	2015	R	45	42	30/15	28/14	9.5±3.9	9.3±3.5
Li et al [24]	2015	R	76	73	51/25	49/24	14.4±6	13.2±7.2
Jiang et al [25]	2016	RCT	74	74	40/34	39/35	33.6±18	34.8±15.6
Liao et al [26]	2016	RCT	30	29	14/16	18/11	12.6±4.92	12.72±2.28
Yang et al [27]	2016	RCT	50	50	36/14	35/15	9.6±2.4	9.9±2.4
Deng et al [28]	2016	RCT	45	45	28/17	30/15	8.8±3.6	8.9±3.8
He et al [29]	2017	RCT	60	60	32/28	31/29	36±18	36±14.4
Xu et al [30]	2017	R	126	120	67/53	65/55	31.2±16.8	32.4±16.8
Zhang et al [31]	2017	RCT	34	34	N	N	N	N
Xie et al [32]	2017	RCT	62	62	40/22	42/20	23.52±6.29	20.67±4.14
Wang et al [33]	2018	R	406	417	298/108	305/112	9.5±1.7	11.3±4.5
Yu et al [34]	2018	R	45	45	22/23	23/22	30.72±7.32	30.72±6.48
Wu et al [35]	2018	RCT	62	62	N	N	N	N
Pan et al [36]	2018	R	373	262	223/150	168/94	13.1±7.3	12.6±6.7
Deng et al [37]	2018	RCT	80	80	61/19	55/25	10.15±4.75	9.93±4.75
Zhang et al [38]	2018	R	45	46	23/22	25/21	3.54±1.44	3.59±1.48
Zhou et al [39]	2019	RCT	41	41	23/18	25/16	10.11±4.15	10.77±4.85
Zhao et al [40]	2019	RCT	37	37	20/17	21/16	10.5±4.8	10.2±5.0
Wang et al [41]	2019	RCT	30	30	21/9	18/12	26.9±19.7	24.8±13.7
Jiang et al [42]	2019	R	58	58	N	N	N	N
Wang et al [43]	2019	RCT	50	50	28/22	27/23	21.48±7.56	17.4±9.96
Zhang et al [44]	2020	R	50	48	37/13	24/14	14.15±6.55	14.57±7.09
Guo et al [45]	2020	R	38	38	20/18	17/21	20.4±13.44	19.8±12.36
Wang et al [46]	2020	R	240	192	N	N	24.00±9.71	20.16±4.10
Li et al [47]	2020	RCT	45	45	28/17	26/19	29.73±7.91	31.24±8.59
Qi et al [48]	2020	RCT	35	35	20/15	21/14	19.08±3.12	18.6±2.76
Sui et al [49]	2021	R	105	104	77/28	68/36	87±13.08	83.64±15.84
Cai et al [50]	2021	RCT	23	22	12/11	12/10	1.62±0.45	1.59±0.45
Ding et al [51]	2021	RCT	31	31	21/10	20/11	15.66±2.73	19.45±2.37
Zhang et al [52]	2021	RCT	76	72	45/31	49/23	42.24±7.32	40.80±6.48
Chen et al [53]	2021	R	42	42	23/19	22/20	11.76±5.04	11.4±4.92
Lian et al [54]	2021	RCT	49	49	27/22	29/20	20.16±6.6	19.92±7.56
Chen et al [55]	2021	RCT	40	40	23/17	24/16	12.36±3.96	12.24±3.96
Du et al [56]	2021	R	45	42	29/16	27/15	13.65±4.27	14.78±5.02
Pei et al [57]	2021	R	43	43	25/18	24/19	22.33±4.55	21.09±4.38
Liu et al [58]	2021	P	1119	1005	731/388	670/335	24.38±23.78	25.80±21.99
Yang et al [12]	2021	R	119	245	89/30	163/82	25.13±2.03	22.47±1.52
Han et al [59]	2022	RCT	90	90	68/22	54/36	8.3±1.6	8.5±1.7
Liu et al [60]	2022	RCT	35	35	20/15	19/16	37.01±3.24	36.01±3.31
Lv et al [61]	2022	R	43	37	30/13	23/14	12.01±1.20	11.82±0.92
Liu et al [62]	2022	RCT	58	58	31/27	30/28	16.23±1.85	15.26±2.05
Pu et al [63]	2022	RCT	75	75	46/29	45/30	12.32±3.15	12.23±3.12
Chukwu et al [64]	2022	RCT	26	26	16/10	19/7	5.5±1.8	6.1±1.6
Lian et al [65]	2023	RCT	40	40	29/11	27/13	13.68±10.01	13.03±7.33

R, retrospective cohort study; RCT, randomized controlled trial study; P, prospective cohort study; N: Not reported; m: Month; U, ultrasound-guided hydrostatic enema reduction; F, fluoroscopy-guided air enema reduction.

**Table 2:** Pooled proportions of clinical characteristics for dichotomous variables (A). Pooled proportions of clinical characteristics for continuous variables (B).

Outcome	Number of studies	Participates (n)		Total number of cases (N)		Statistical results			Heterogeneity		Analysis model
		U	F	U	F	Statistic	Value(95%CI)	P value	I^2^ (%)	P value	
Male	45	2839	2712	4447	4204	RR	0.994(0.964,1.026)	0.718	0.00	0.988	Fixed
Female	45	1608	1492	4447	4204	RR	1.010(0.955,1.069)	0.720	0.00	0.993	Fixed
Paroxysmal crying or Abdominal pain	18	1446	1322	1741	1616	RR	1.031(0.995,1.068)	0.096	32.30	0.098	Fixed
Vomiting	17	1335	1216	1718	1594	RR	0.969(0.928,1.011)	0.149	0.00	0.624	Fixed
Abdominal mass	13	590	692	820	935	RR	1.007(0.938,1.081)	0.852	0.00	0.594	Fixed
Bloody stool	17	622	632	1696	1571	RR	0.963(0.855,1.085)	0.536	55.80	0.003	Random
Success rate of reset	48	4518	3766	4722	4305	RR	1.128(1.099,1.157)	<0.001^※^	71.40	<0.001^※^	Random
Recurrence	25	186	293	3134	2680	RR	0.391(0.269,0.569)	<0.001^※^	51.50	0.002	Random
Total complications	20	58	195	1349	1225	RR	0.296(0.225,0.389)	<0.001^※^	13.30	0.288	Fixed
Perforation	23	13	43	2376	2381	RR	0.405(0.244,0.670)	<0.001^※^	0.00	0.968	Fixed
Vomiting	10	14	32	619	563	RR	0.463(0.271,0.791)	0.050	0.00	0.825	Fixed
Diarrhea	9	13	43	558	507	RR	0.318(0.182,0.558)	<0.001^※^	0.00	0.948	Fixed

Outcome	Number of studies	Total number of cases (N)		Statistical results			Heterogeneity		Analysis model
		U	F	Statistic	Value(95%CI)	P value	I^2^ (%)	P value	
Age	45	4335	4166	WMD	0.379(-0.128,0.885)	0.143	90.00	<0.001^※^	Random
Duration of onset	29	1961	1780	WMD	-0.296(-1.788,1.197)	0.698	97.00	<0.001^※^	Random
Resetting time	31	2146	2090	WMD	-4.183(-5.402,-2.964)	<0.001^※^	98.60	<0.001^※^	Random
Resetting pressure	4	234	360	WMD	1.550(-0.292,3.392)	0.099	99.80	<0.001^※^	Random
Duration of occult blood in stool	7	435	431	WMD	-0.808(-1.098,-0.517)	<0.001^※^	89.70	<0.001^※^	Random
Length of hospitalization	18	1772	1780	WMD	-1.215(-1.58,-0.85)	<0.001^※^	99.40	<0.001^※^	Random

RR, relative risk; CI, confidence interval; U, ultrasound-guided hydrostatic enema reduction; F, fluoroscopy-guided air enema reduction; ^※^, P < 0.05 was considered to be statistically significant.

WMD, weighted mean difference; CI, confidence interval; U, ultrasound-guided hydrostatic enema reduction; F, fluoroscopy-guided air enema reduction; ^※^, P < 0.05 was considered to be statistically significant.

### Comparations and outcomes of the meta-analysis

#### Age of operation

Forty-five studies contributed data about UGHR and FGAR, including 8501 patients (4335 in the UGHR and 4166 in the FGAR, Table 2(B)). Random-effects model was applied because of significant heterogeneity (I^2^ = 90.00%, P < 0.001 Table 2(B)). Meta-analysis showed no significant difference between the two groups [WMD = 0.379, 95% CI = (-0.128,0.885), P = 0.143 > 0.05].

#### Duration of onset

Twenty-nine studies contributed data about UGHR and FGAR, including 3741 patients (1961 in the UGHR and 1780 in the FGAR, Table 2(B)). Random-effects model was applied because of significant heterogeneity (I^2^ = 97.00%, P < 0.001 Table 2(B)). Meta-analysis showed no significant difference between the two groups [WMD = -0.296, 95% CI = (-1.788,1.197), P = 0.698 > 0.05].

#### Clinical symptoms

Clinical symptoms reported in the studies primarily encompassed paroxysmal crying or abdominal pain, vomiting, the presence of an abdominal mass, and the passage of bloody stools.

Paroxysmal crying or abdominal pain: Eighteen studies contributed data about UGHR and FGAR, including 2768 patients (1446/1741 in the UGHR and 1322/1616 in the FGAR, Table 2(A)). Fixed-effects model was applied because of low heterogeneity (I^2^ = 32.30%, P = 0.098 Table 2(A)). Meta-analysis showed no significant difference between the two groups [RR = 1.031, 95% CI = (0.995,1.068), P = 0.096 > 0.05].

Vomiting: Seventeen studies contributed data about UGHR and FGAR, including 2551 patients (1335/1718 in the UGHR and 1216/1594 in the FGAR, Table 2(A)). Fixed-effects model was applied because of low heterogeneity (I^2^ = 0.00%, P = 0.624 Table 2(A)). Meta-analysis showed no significant difference between the two groups [RR = 0.969, 95% CI = (0.928,1.011), P = 0.149 > 0.05].

Abdominal mass: Thirteen studies contributed data about UGHR and FGAR, including 1282 patients (590/820 in the UGHR and 692/935 in the FGAR, Table 2(A)). Fixed-effects model was applied because of low heterogeneity (I^2^ = 0.00%, P = 0.594 > 0.05 Table 2(A)). Meta-analysis showed no significant difference between the two groups [RR = 1.007, 95% CI = (0.938,1.081), P = 0.852 > 0.05].

Bloody stool: Seventeen studies contributed data about UGHR and FGAR, including 1254 patients (622/1696 in the UGHR and 632/1571 in the FGAR, Table 2(A)). Random-effects model was applied because of significant heterogeneity (I^**2**^ = 55.80%, P = 0.003 Table 2(A)). Meta-analysis showed no significant difference between the two groups [RR = 0.963, 95% CI = (0.855,1.085), P = 0.536 > 0.05].

#### Outcomes

The primary outcome measures for enema reduction in cases of intussusception comprise resetting time, resetting pressure, success rate of reduction, duration of occult blood in stool, length of hospitalization, and recurrence.

Resetting time: Thirty-one studies contributed data about UGHR and FGAR, including 4236 patients (2146 in the UGHR and 2090 in the FGAR, Table 2(B)). Random-effects model was applied because of significant heterogeneity (I^2^ = 98.60%, P < 0.001 Table 2(B)). Meta-analysis showed significant difference between the two groups [WMD = -4.183, 95% CI = (-5.402, -2.964), P < 0.001; S2 Fig], which demonstrated significantly less resetting time of UGHR.

Resetting pressure: Four studies contributed data about UGHR and FGAR, including 594 patients (234 in the UGHR and 360 in the FGAR, Table 2(B)). Random-effects model was applied because of significant heterogeneity (I^2^ = 99.80%, P < 0.001 Table 2(B)). Meta-analysis showed significant difference between the two groups [WMD = 1.55, 95% CI = (-0.292,3.392), P = 0.099 > 0.05], which demonstrated significantly less resetting time of UGHR.

Success rate of reset: Forty-eight studies contributed data about UGHR and FGAR, including 8284 patients (4518/4722 in the UGHR and 3766/4305 in the FGAR, Table 2(A)). Random-effects model was applied because of significant heterogeneity (I^2^ = 71.40%, P < 0.001 Table 2(A)). Meta-analysis showed significant difference between the two groups [RR = 1.128, 95% CI = (1.099,1.157), P < 0.001; S3 Fig], which demonstrated significantly higher reset success rate of UGHR.

Duration of occult blood in stool: Seven studies contributed data about UGHR and FGAR, including 866 patients (435 in the UGHR and 431 in the FGAR, Table 2(B)). Random-effects model was applied because of significant heterogeneity (I^2^ = 89.70%, P < 0.001 Table 2(B)). Meta-analysis showed significant difference between the two groups [WMD = -0.808, 95% CI = (-1.098, -0.517), P < 0.001], which demonstrated significantly shorter duration of occult blood in stool of UGHR.

Length of hospitalization: Eighteen studies contributed data about UGHR and FGAR, including 3552 patients (1772 in the UGHR and 1780 in the FGAR, Table 2(B)). Random-effects model was applied because of significant heterogeneity (I^2^ = 99.40%, P < 0.001 Table 2(B)). Meta-analysis showed significant difference between the two groups [WMD = -1.215, 95% CI = (-1.58, -0.85), P < 0.001; S4 Fig], which demonstrated significantly shorter length of hospitalization of UGHR.

Recurrent rate: Twenty-five studies contributed data about UGHR and FGAR, including 479 patients (186/3134 in the UGHR and 293/2680 in the FGAR, Table 2(A)). Random-effects model was applied because of significant heterogeneity (I^2^ = 51.50%, P < 0.001 Table 2(A)). Meta-analysis showed significant difference between the two groups [RR = 0.391, 95% CI = (0.269,0.569), P = 0.002<0.05; S5 Fig], which demonstrated significantly less relapse rate of UGHR.

### Complications

To describe the occurrence of complications during the enema reduction procedure for intussusception, we calculated the overall complication rate, perforation rate, as well as rates of vomiting and diarrhea.

Total complications rate: Twenty studies contributed data about UGHR and FGAR, including 253 patients (58/1349 in the UGHR and 195/1225 in the FGAR, Table 2(A)). Fixed-effects model was applied because of low heterogeneity (I^2^ = 13.30%, P = 0.288 Table 2(A)). Meta-analysis showed significant difference between the two groups [RR = 0.296, 95% CI = (0.225,0.389), P < 0.001; S6 Fig], which demonstrated significantly lower total complications rate of UGHR.

Perforation rate: Twenty-three studies contributed data about UGHR and FGAR, including 56 patients (13/ 2376 in the UGHR and 43/2381 in the FGAR, Table 2(A)). Fixed-effects model was applied because of low heterogeneity (I^2^ = 0.00%, P = 0.968 Table 2(A)). Meta-analysis showed significant difference between the two groups [RR = 0.405, 95% CI = (0.244,0.670), P < 0.001; S7 Fig], which demonstrated significantly lower perforation rate of UGHR.

Incidence of post-operative vomiting: Ten studies contributed data about UGHR and FGAR, including 46 patients (14/619 in the UGHR and 32/563 in the FGAR, Table 2(A)). Fixed-effects model was applied because of low heterogeneity (I^2^ = 0.00%, P = 0.825 Table 2(A)). Meta-analysis showed significant difference between the two groups [RR = 0.463 , 95% CI = (0.271,0.791), P < 0.001], which demonstrated significantly lower post-operative vomiting rate of UGHR.

Incidence of post-operative diarrhea: Nine studies contributed data about UGHR and FGAR, including 56 patients (13/ 558 in the UGHR and 43/507 in the FGAR, Table 2(A)). Fixed-effects model was applied because of low heterogeneity (I^2^ = 0.00%, P = 0.948 Table 2(A)). Meta-analysis showed significant difference between the two groups [RR = 0.318 , 95% CI = (0.182,0.558), P < 0.001], which demonstrated significantly lower post-operative diarrhea rate of UGHR.

### Publication bias

Begg’s Test and Egger’s Test were performed, and Begg’s funnel plots were generated for some of the included records. Different subgroups were defined to assess publication bias (Table 3). Several largely symmetrical inverted funnel plots were observed (S8A–S8D Fig), and publications displaying significant bias were removed.

**Table 3:** Begg’s and Egger’s test of publication bias of clinical characteristics.

Outcome	Number of studies	P-value^a^	
		Begg’test	Egger’test
**Gender**			
Male	45	0.883	0.388
Female	45	0.604	0.117
Age	45	0.087	0.893
Duration of onset	29	0.003*	0.485
**Clinical symptoms**			
Paroxysmal crying or abdominal pain	18	0.127	0.025
Vomiting	17	0.753	0.462
Abdominal mass	13	1.000	0.607
Bloody stool	17	0.174	0.249
**Ending indicators**			
Resetting time	31	0.248	0.004*
Resetting pressure	4	0.734	0.378
Success rate of reset	48	0.001*	0.000*
Duration of occult blood in stool	7	0.764	0.811
Length of hospitalization	18	0.069	0.676
Recurrence	25	0.216	0.618
**Complications**			
Total complications	20	0.456	0.845
Perforation	23	0.128	0.236
Vomiting	10	0.371	0.795
Diarrhea	9	0.348	0.166

^**a**^: P value means the value of Pr>|z| (continuity corrected, in Begg’s Test) or P>|t| (in Egger’s Test)

^*^:P value < 0.05 was considered to have a high risk of publication bias.

## Discussions

Pediatric intussusception is characterized by the invagination of one segment of the bowel into an immediately adjacent segment, resulting in the obstruction of intestinal contents. Over time, compromised vascular flow to the affected segment can lead to ischemia, necrosis, and potentially perforation [10,66]. Therefore, early diagnosis and prompt treatment are imperative to improve prognosis. While radiological imaging plays a pivotal role in diagnosing and treating this condition, it is often not the initial choice in clinical practice due to concerns regarding radiation exposure. Ultrasound, conversely, stands out as the preferred imaging modality for diagnosis owing to its remarkable specificity (88%-100%), high sensitivity (98%-100%), and absence of ionizing radiation [67–69]. In cases of uncomplicated pediatric intussusception, imaging-guided enema reduction stands as the globally recognized standard for nonsurgical treatment [70]. To evaluate the efficacy and safety of ultrasound-guided hydrostatic enema reduction (UGHR) versus fluoroscopy-guided air enema reduction (FGAR), we conducted a comprehensive analysis encompassing clinical presentations, outcome parameters, and postoperative complications in both groups. Our primary objective is to equip healthcare practitioners with valuable insights for making informed treatment decisions when managing patients with intussusception.

We enrolled a total of 49 studies into our analysis, of which was based on a mixture of randomized and non-randomized trials. The outcomes of the meta-analysis concerning clinical presentations of intussusception, including paroxysmal crying and abdominal pain, the presence of an abdominal mass, time of onset and the occurrence of blood in stools, consistently indicated no significant differences when comparing the two groups.

The findings of this meta-analysis indicate that UGHR is characterized by a shorter resetting time, a higher success rate of reset, and a reduced duration of hospitalization (Table 2A and 2B). It has been proposed that during the UGHR procedure, real-time ultrasound enables the observation of the gradual movement of the intussusception towards the ileocecal region. During this phase, increasing the enema pressure can enhance the repositioning success rate and decrease the repositioning time. Additionally, the use of warm saline aids in the expulsion of intestinal contents, reducing the absorption of toxins by the intestinal tract. This, in turn, mitigates complications in children following the enema reduction, ultimately leading to a shorter hospital stay [12,36,41,62,71].

Complications arising from intussusception enema reduction are a critical aspect of assessing its safety, with intestinal perforation being one of the most severe complications [72]. During air enema, when the intestinal lumen pressure is high, the intestinal tube undergoes significant expansion. If excessive or sudden pressure is applied, air entering the terminal ileum may result in a tense pneumoperitoneum, potentially leading to intestinal perforation [73]. It has been reported [74] that UGHR may be less hygienic and could lead to intra-abdominal fecal contamination in case of intestinal perforation, which, if not promptly treated, can result in severe complications and endanger the patient’s life. The meta-analysis presented in this article demonstrates that UGHR repositioning is associated with a lower overall complication rate, including a lower incidence of perforation (Table 2A). Furthermore, the occurrence of postoperative vomiting and diarrhea is significantly reduced in children. Pan et al [36] suggest that this reduction in complications may be attributed to the slower movement of the water column during the water enema, causing less damage to the intestinal mucosa and possessing some mucosal dialysis function, resulting in a lower incidence of postoperative complications. Additionally, UGHR enables the measurement of intestinal tube hemodynamics, observation of the intestinal wall’s blood supply, and determination of its viability. This can effectively mitigate the risk of perforation due to high pressure during the enema procedure [41,63]. It is recommended to employ intermittent ultrasound monitoring to assess the intestinal canal diameter during enema operations, reducing the likelihood of perforation. UGHR also allows for clear visualization of the intussusception mass and early detection of pathological predisposing points or residual intussusception. Overall, it can be inferred that UGHR provides significant advantages in the treatment of intussusception in children.

However, the main disadvantage of UGHR is that the success of its enemas is significantly related to the experience of the operator, which requires pediatric surgeons to be taught and trained in ultrasound or radiology. This study exhibits several limitations too. Firstly, it’s worth noting that most studies included in this analysis were single-center trials. While our overall sample size is substantial, single-center studies may induce inevitable biases. Secondly, it’s noteworthy that the surgical team was also involved in authoring the reports. This potential author-surgeon bias should be taken into consideration when interpreting the findings. Thirdly, certain outcome measures, such as repositioning pressure, duration of postoperative blood in the stool, and postoperative vomiting or diarrhea, exhibited lower reliability due to a limited number of reported studies, resulting in a relatively small sample size for these specific parameters. Lastly, the enrolled studies were mostly from the Asia and Europe, an inevitable selection bias was existed.

## Conclusions

In conclusion, it can be affirmed that both UGHR and FGAR represent safe and effective nonsurgical approaches for the management of pediatric acute intussusception. However, when comparing the two methods, UGHR emerges as the preferable choice. This preference is rooted in its nonradioactive nature, quicker repositioning times, higher success rates in repositioning, reduced postoperative hospitalization durations, fewer overall postoperative complications, and a notably decreased incidence of concurrent perforation when compared to FGAR.

## Supporting information

S1 Checklist PRISMA 2020 checklist. (DOCX)

S1 Fig A. Risk of bias summary graph 1 for the included randomized controlled trial. B. Risk of bias summary graph 2 for the included randomized controlled trial.
(TIF)

S2 Fig Comparations of resetting time in ultrasound-guided hydrostatic enema reduction (UGHR) and fluoroscopy-guided air enema reduction (FGAR). (TIF)

S3 Fig Comparations of the rate of successful reset in ultrasound-guided hydrostatic enema reduction (UGHR) and fluoroscopy-guided air enema reduction (FGAR). (TIF)

S4 Fig Comparations of the length of hospitalization in ultrasound-guided hydrostatic enema reduction (UGHR) and fluoroscopy-guided air enema reduction (FGAR). (TIF)

S5 Fig Comparations of the rate of recurrent in ultrasound-guided hydrostatic enema reduction (UGHR) and fluoroscopy-guided air enema reduction (FGAR). (TIF)

S6 Fig Comparations of the rate of total complications in ultrasound-guided hydrostatic enema reduction (UGHR) and fluoroscopy-guided air enema reduction (FGAR). (TIF)

S7 Fig Comparations of the rate of perforation in ultrasound-guided hydrostatic enema reduction (UGHR) and fluoroscopy-guided air enema reduction (FGAR). (TIF)

S8 Fig A. Meta-analysis of male between UGHR and FGAR. B. Meta-analysis of the rate of perforation between UGHR and FGAR. C. Meta-analysis of vomiting between UGHR and FGAR. D. Meta-analysis of age between UGHR and FGAR.
(TIF)

S1 Table Newcastle-Ottawa scale scores for non-randomized controlled studies. (DOCX)

S1 Dataset Minimal dataset underlying the results. (XLSX)
