# Supplementary figures and images for: Air enema reduction versus hydrostatic enema reduction for intussusceptions in children: A systematic review and meta-analysis
Source: PLoS One. 2024 Mar 18;19(3):e0297985. doi: 10.1371/journal.pone.0297985 (PMC10947698; doi:10.1371/journal.pone.0297985)

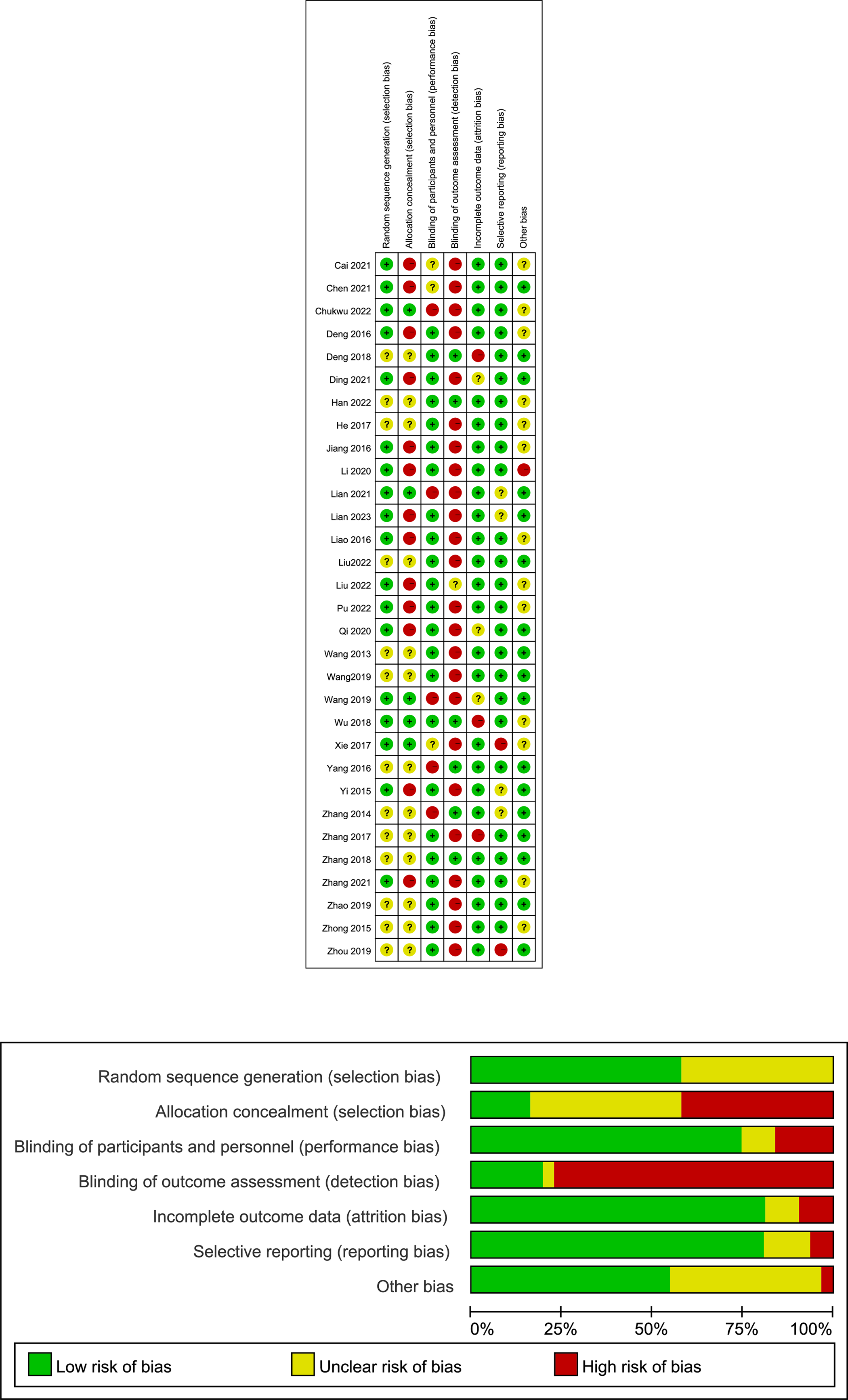

Supplement: S1 Fig — A. Risk of bias summary graph 1 for the included randomized controlled trial. B. Risk of bias summary graph 2 for the included randomized controlled trial. (TIF) [file pone.0297985.s002.tif]

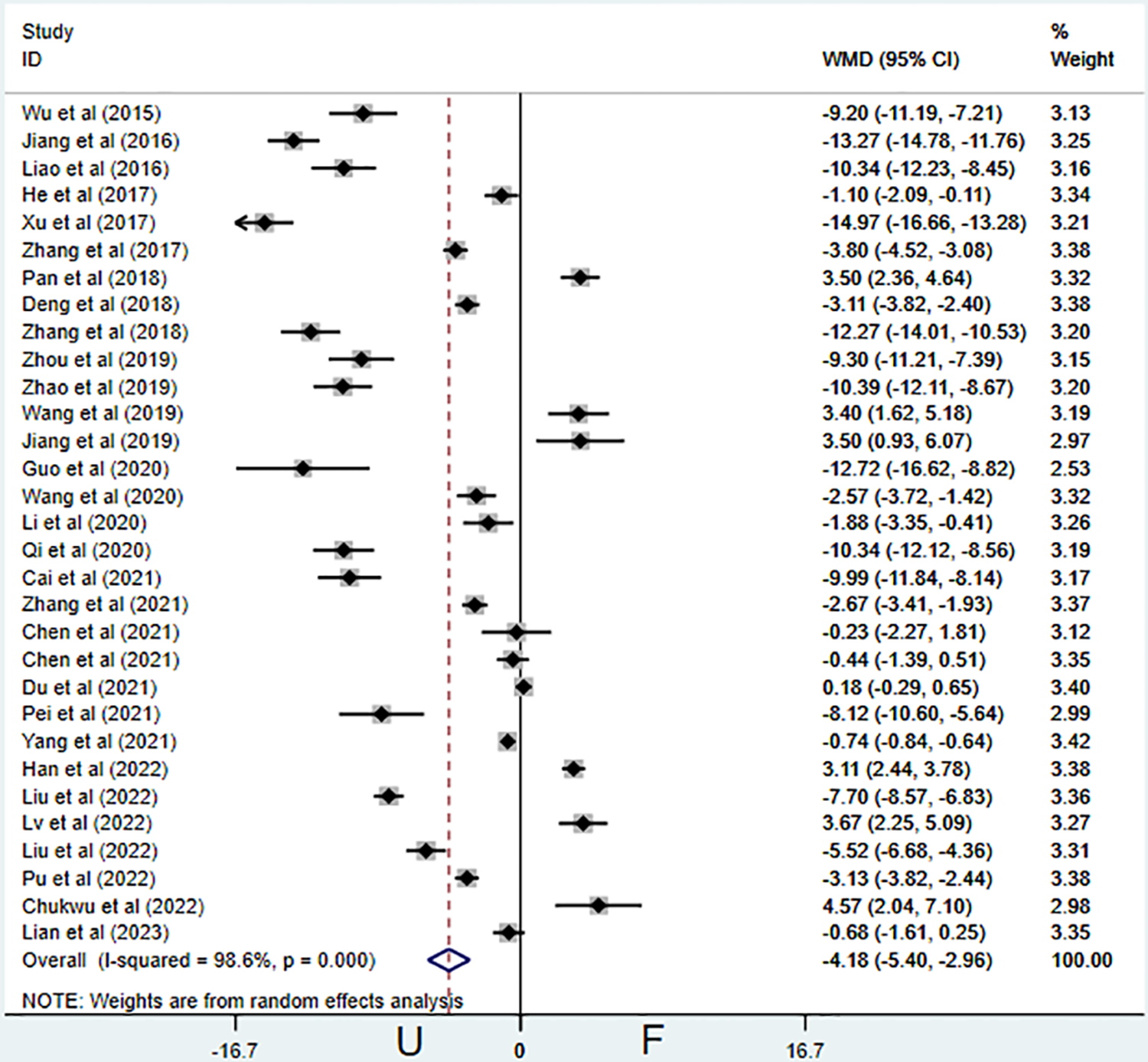

Supplement: S2 Fig — (TIF) [file pone.0297985.s003.tif]

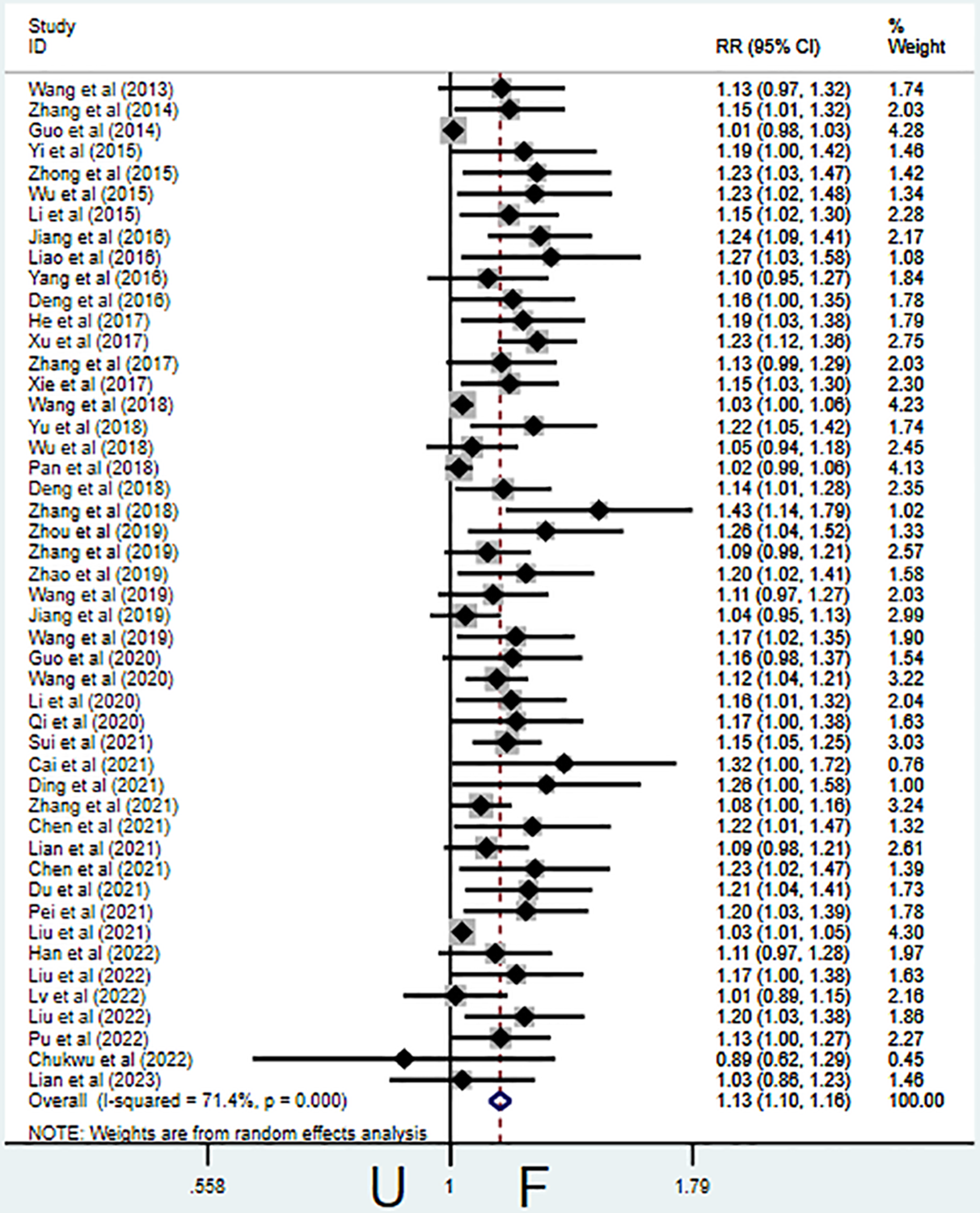

Supplement: S3 Fig — (TIF) [file pone.0297985.s004.tif]

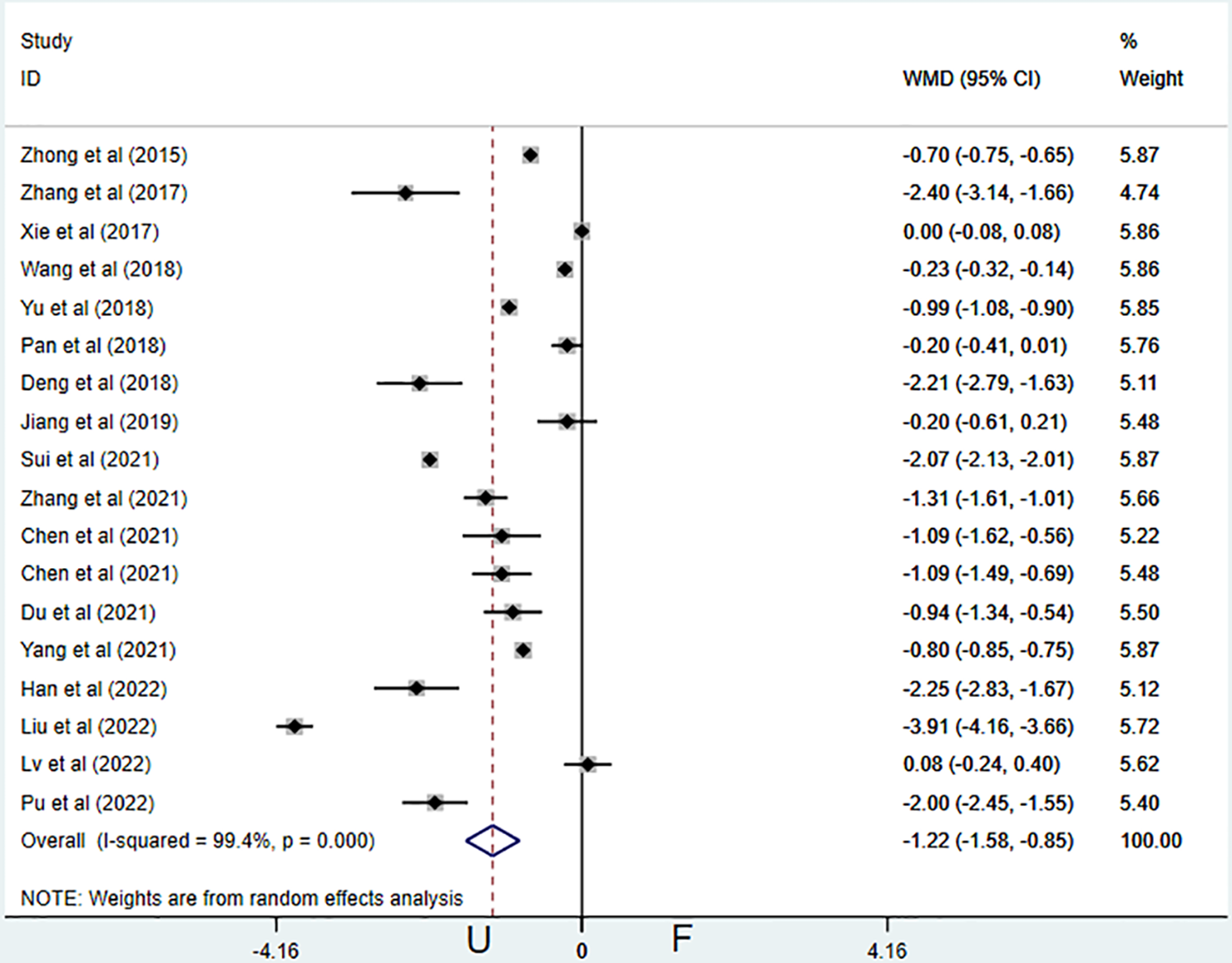

Supplement: S4 Fig — (TIF) [file pone.0297985.s005.tif]

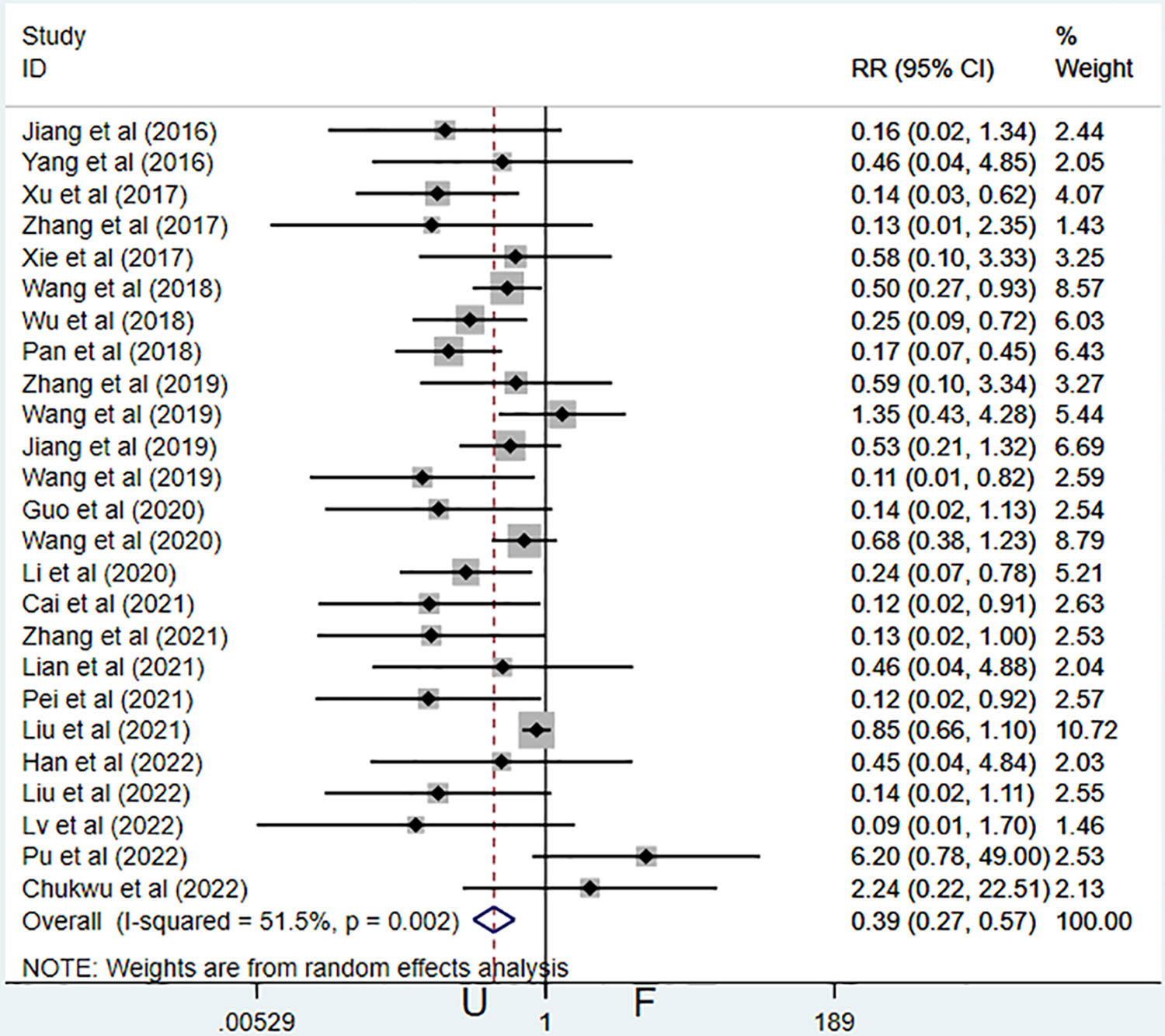

Supplement: S5 Fig — (TIF) [file pone.0297985.s006.tif]

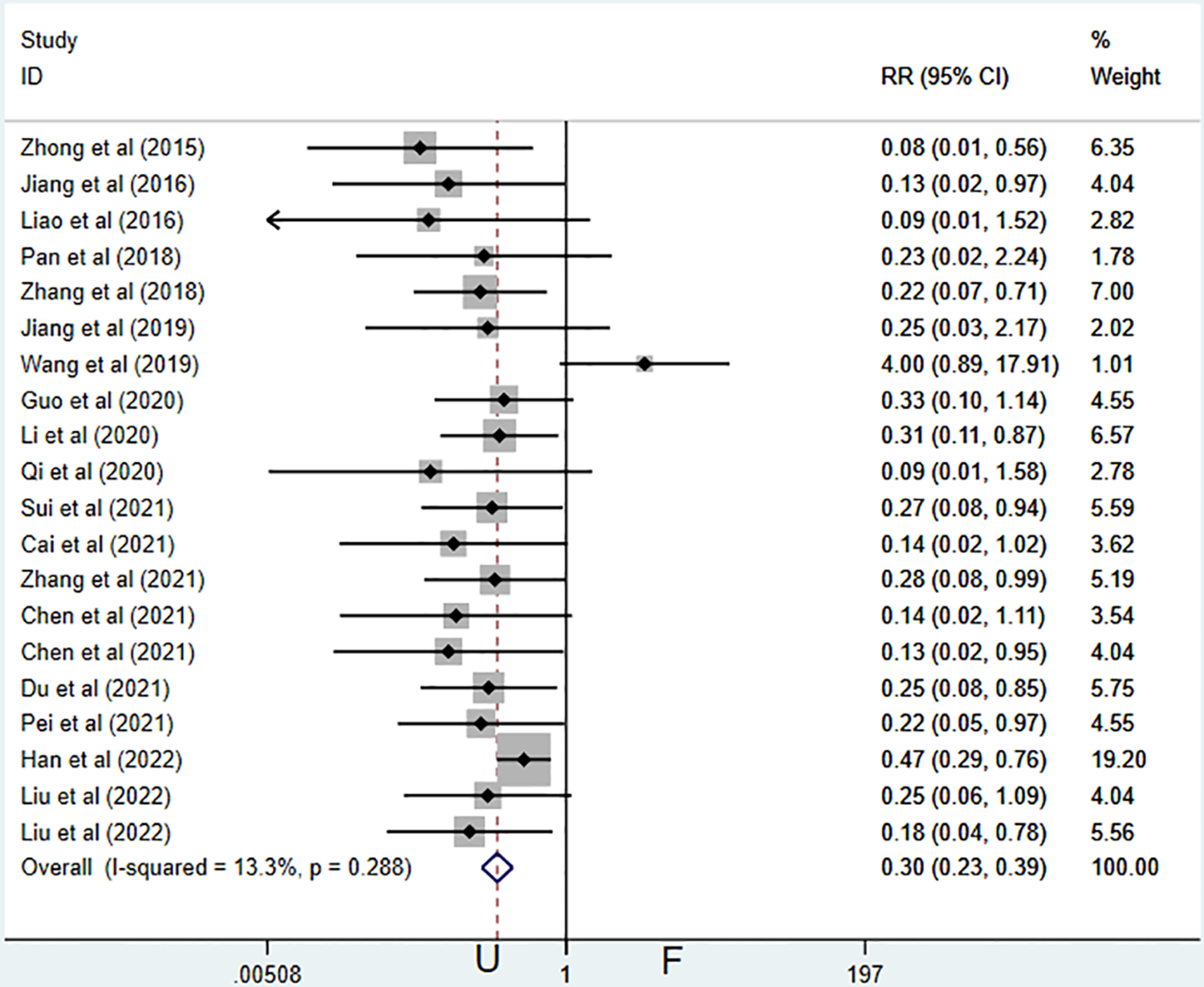

Supplement: S6 Fig — (TIF) [file pone.0297985.s007.tif]

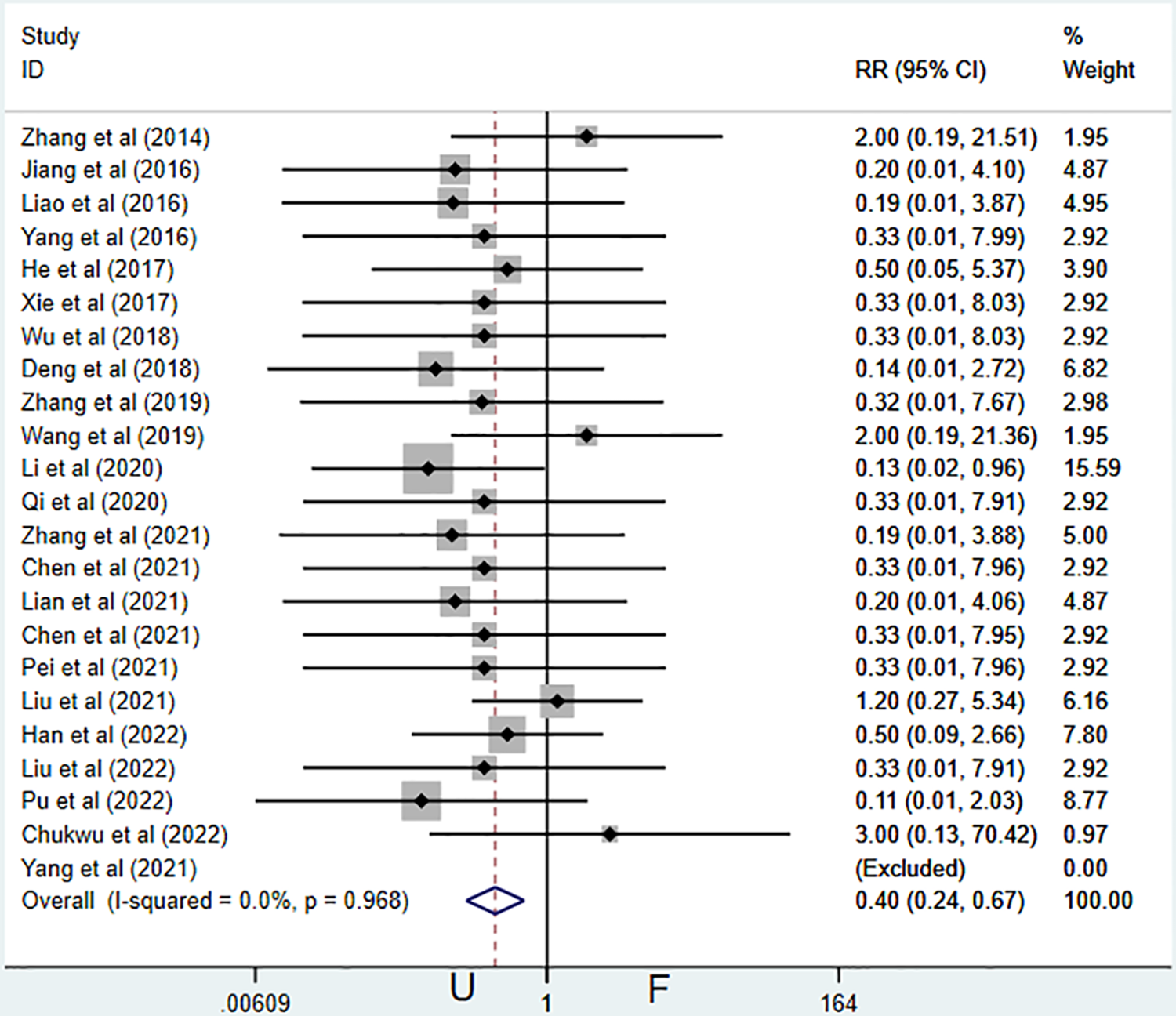

Supplement: S7 Fig — (TIF) [file pone.0297985.s008.tif]

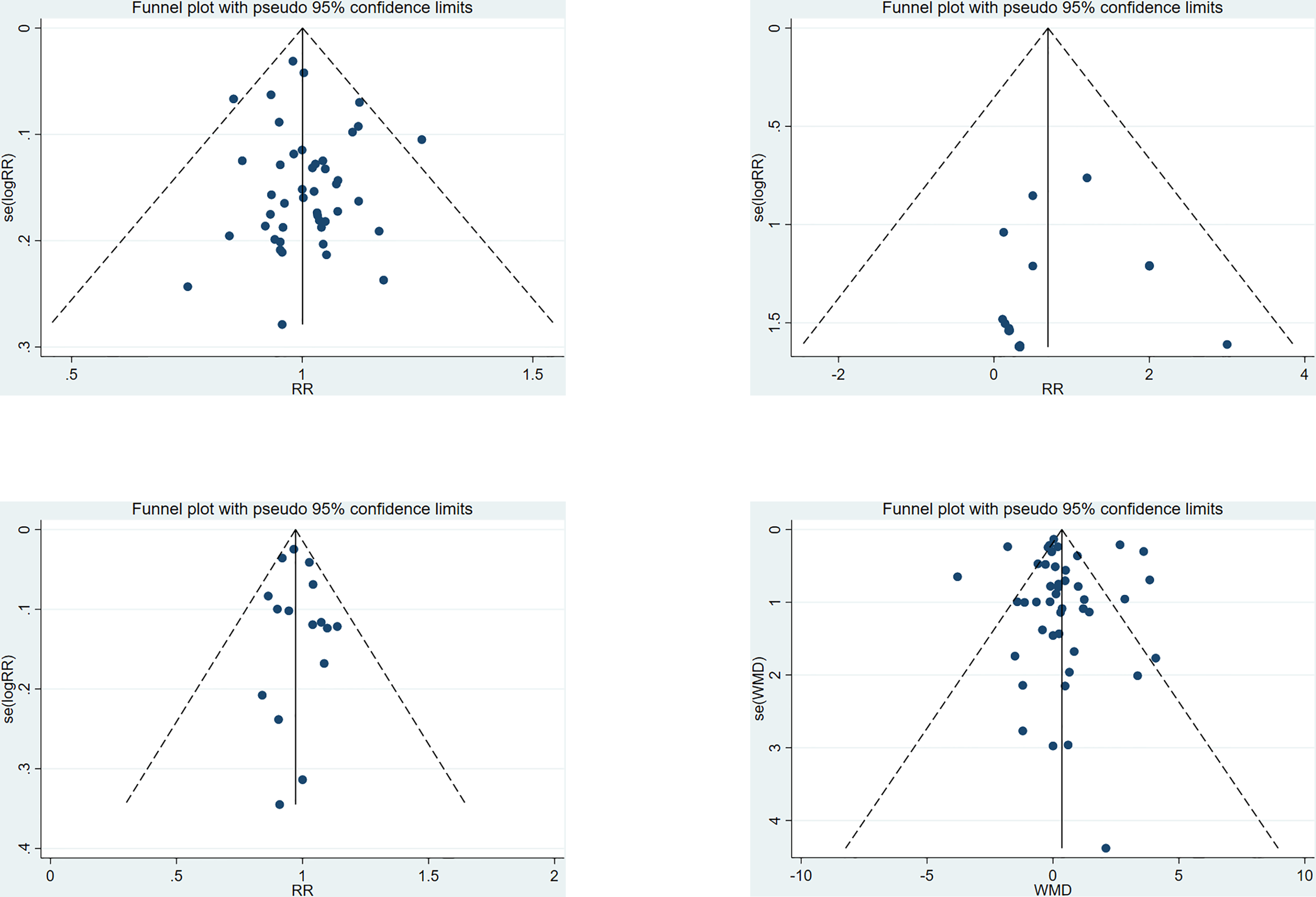

Supplement: S8 Fig — A. Meta-analysis of male between UGHR and FGAR. B. Meta-analysis of the rate of perforation between UGHR and FGAR. C. Meta-analysis of vomiting between UGHR and FGAR. D. Meta-analysis of age between UGHR and FGAR. (TIF) [file pone.0297985.s009.tif]
