# Supplementary material for: Air enema reduction versus hydrostatic enema reduction for intussusceptions in children: A systematic review and meta-analysis
Source: PLoS One. 2024 Mar 18;19(3):e0297985. doi: 10.1371/journal.pone.0297985 (PMC10947698; doi:10.1371/journal.pone.0297985)
Supplement: S1 Table — (DOCX) [file pone.0297985.s010.docx]

**S1 Table.** Newcastle-Ottawa Scale scores for non-randomized controlled studies.

| Study | Selection | Comparability | Outcome/Exposure | Total |
| --- | --- | --- | --- | --- |
| Guo et al[20] | 3 | 1 | 2 | 6 |
| Wu et al[23] | 3 | 2 | 3 | 8 |
| Li et al[24] | 3 | 2 | 2 | 7 |
| Xu et al[30] | 3 | 2 | 2 | 7 |
| Wang et al[33] | 3 | 2 | 2 | 7 |
| Yu et al[34] | 3 | 2 | 2 | 7 |
| Pan et al[36] | 3 | 2 | 3 | 8 |
| Jiang et al[42] | 3 | 2 | 3 | 8 |
| Zhang et al[44] | 3 | 2 | 2 | 7 |
| Guo et al[45] | 3 | 2 | 3 | 8 |
| Wang et al[46] | 3 | 2 | 3 | 8 |
| Sui et al[49] | 3 | 2 | 2 | 7 |
| Chen et al[53] | 3 | 2 | 3 | 8 |
| Du et al[56] | 3 | 2 | 3 | 8 |
| Pei et al[57] | 3 | 2 | 3 | 8 |
| Liu et al[58] | 3 | 2 | 3 | 8 |
| Yang et al[12] | 3 | 2 | 3 | 8 |
| Lv et al[61] | 3 | 2 | 3 | 8 |
