# Supplementary material for: Air enema reduction versus hydrostatic enema reduction for intussusceptions in children: A systematic review and meta-analysis
Source: PLoS One. 2024 Mar 18;19(3):e0297985. doi: 10.1371/journal.pone.0297985 (PMC10947698; doi:10.1371/journal.pone.0297985)
Supplement: Attachment — Submitted filename: Response to Reviewers.docx [file pone.0297985.s012.docx]

**Point-by-point response to edit's comments**

\# ACADEMIC EDITOR

Q 1: The methodology needs to be revised. The details of search strategy, definition of PICOS, the inclusion and exclusion criteria should be addressed more clearly for the readers. In addition, the selection process and data extraction process should be given clearly.

A 1: Thank you very much for your valuable suggestions, I will answer your questions in detail gladly.

**Detailed part of the search strategy:** we formulated the search strategy, inclusion criteria and exclusion criteria according to the PICOS principles: (1) Type of study: randomized or non-randomized controlled trial, with the language limited to Chinese and English; (2) Participants of the study: pediatric patients (aged <18 years) who underwent enemas due to intussusception; (3) Interventions adopted: fluoroscopic air enema or ultrasound-guided saline enemas were used; (4) The main outcome indicators: time to reset, success rate of reset, recurrence rate, and occurrence of postoperative complications. (5) Search strategy: Take Pubmed as an example: a specific search strategy is shown (see the suppl-figure 1 and revised-Figure 1 in the manuscript), and we have revised the text in red (page 5, line 2-9).

**Literature search and data collection part:** The literature retrieval and data collection are to be carried out by at least two researchers. They will independently read the titles and abstracts of the literature, excluding those that are not relevant to the content of this study. Subsequently, they will carefully read the full texts according to inclusion and exclusion criteria, extracting relevant information. In case of disagreements, resolution will be sought through negotiation, or a third researcher may be consulted for assistance in making a judgment. (page 6, line 21-22, page 7, line 1-6)

Q 2: Regarding screening process, the reason for exclusion of the studies should be given clearly in order to prevent bias while interpreting the results.

A 2: I am so grateful for the valuable advice, the literature screening process involved 2 researchers independently reading the titles and abstracts of the literature to initially exclude literature that did not match the content of this study for the specific reasons for exclusion shown in revised-Figure 1 in the manuscript

Q 3: When interpreting the results, it will be better to comment on the distribution of the countries in which the included studies were published.

A 3: Thank you for this valuable suggestion. We have added associated information in the result and the limitations.

Q 4: Regarding the funding, the authors stated that there is no funding during submission, they stated that there is funding in the end of the article. Please verify clearly.

A 4: I am very sorry for your misunderstanding due to my negligence during submission, this article does have financial support and we will make changes subsequently.

\# Reviewer 1

Q 1: Title: The title is better as “Air enema reduction versus hydrostatic enema reduction for intussusceptions in children: A systematic review and meta-analysis”. Avoid such terms as updated or comprehensive. Meta-analysis is expected to be comprehensive and to provide updated information

A1: Thank you very much for your valuable suggestions, the title of this article has been changed. (see the title)

Q 2: Introduction: There is need for more details on rationale for the study, a study question, and a hypothesis.

A2: Thank you very much for your valuable suggestions, we have revised the article's introduction. (see the introduction in red）

Q 3: Methods: Was any article excluded based on the language of publication? How many authors independently reviewed the article from the initial search, and critical review of included articles? How did the authors address inter-rater variability in the review of included articles?

A 3: I am so grateful for the valuable advice. We formulated the search strategy, inclusion criteria and exclusion criteria according to the PICOS principles: (1) Type of study: randomized or non-randomized controlled trial, with the language limited to Chinese and English; (2) Participants of the study: pediatric patients (aged <18 years) who underwent enemas due to intussusception; (3) Interventions adopted: fluoroscopic air enema or ultrasound-guided saline enemas were used; (4) The main outcome indicators: time to reset, success rate of reset, recurrence rate, and occurrence of postoperative complications. (page 5, line 2-9)

The literature retrieval and data collection are to be carried out by at least two researchers. They will independently read the titles and abstracts of the literature, excluding those that are not relevant to the content of this study. Subsequently, they will carefully read the full texts according to inclusion and exclusion criteria, extracting relevant information. In case of disagreements, resolution will be sought through negotiation, or a third researcher may be consulted for assistance in making a judgment. (page 6, line 21-22, page 7, line 1-6)

Q 4: Discussion: Repeat of information already in the methods and results sections. I would like a succinct summary of your main findings.

A4: Thank you very much for your valuable suggestions, in the discussion section of the article, we focus on statistically significant indicators and discuss the reasons for such results in the context of the clinic, so it is not concise enough, and We have also made certain modifications. (see the discussion in red)

Q 5: References: The referencing style is not uniform.

A4: Please accept my sincere thanks for your help with my article regarding the revision of the references, which I have accepted and revised. (see the references)

\# Reviewer 2

 Q 1: The authors review the two methods of reduction in the introduction and discussion. As noted above, the included literature in the search seems to be a sub-selection without appropriate screening of all literature identified by the initial search. I would request clarification regarding the 986 articles that were identified but expunged prior to screening. I would suggest the authors screen these 986 articles using their inclusion and exclusion criteria to ensure all relevant literature is considered.

A1: Please accept my sincere thanks for your help with my article, we developed inclusion and exclusion criteria based on the PICOS principles and conducted a comprehensive literature search in which two investigators independently read article titles and abstracts to initially exclude literature that did not match the content of this study, with specific reasons for exclusion demonstrated (see the suppl-figure 1 and revised-Figure 1 in the manuscript), and we have revised the text in red (page 5, line 2-9).

Q 2: It is helpful that the authors have assessed the risk of bias for individual studies. I would consider highlighting in the discussion that the literature is based on a mix of randomized and non-randomized trials. Specifically, the fact that one very large retrospective cohort study contributes 1119 patients to the total of 9391 (and most of the included randomized trials are much smaller than the non-randomized). Although this information is available in Table 1, I would suggest making it more accessible to readers.

A2: I am so grateful for the valuable advice, we have made changes in the discussion section and added that element. (page 15, line 14-15)

Q 3: Overall this is a well-written manuscript and the comparison of UGHR and FGAR is worthwhile; however, revision is required to include screening of all appropriate literature as there may be selection bias with the current methods that do not explain why only a subset of literature underwent screening.

A 3: Thank you very much for your valuable suggestions, As described in the text, our literature search strategy was to abstract or title all literature including "intussusception", "enema", "ultrasound-guided", so by reading the title and abstract of those articles, it was possible to exclude most of the literature. For example, studies in which the subject was not a child, uncontrolled studies (only UGHR or FGAR), or fluoroscopic or ultrasound-guided enemas with other fluid will be excluded after screening. (see the suppl-figure 1 and revised-Figure 1 in the manuscript)

#1 intussusceptions [Mesh]

#2 ( (enema [Title/Abstract]) AND (intussusceptions [Title/Abstract]))

#3 hydrostatic enema for intussusceptions

#4 ((enema[Title/Abstract]) AND (intussusceptions[Title/Abstract])) AND (ultrasound[Title/Abstract])

#5 (intussusceptions [Title/Abstract])) AND (ultrasound) [Title/Abstract])

#6 #1 OR #2

#7 #1 OR #3

#8 #1 OR #4

#9 #1 OR #5

#10 #6 OR #7 OR #8 OR #9

**Suppl-Figure1** Take PubMed as an example to show a specific search strategy of UGHR.
